# Coumarin Reduces Virulence and Biofilm Formation in *Pseudomonas aeruginosa* by Affecting Quorum Sensing, Type III Secretion and C-di-GMP Levels

**DOI:** 10.3389/fmicb.2018.01952

**Published:** 2018-08-21

**Authors:** Yunhui Zhang, Andrea Sass, Heleen Van Acker, Jasper Wille, Bruno Verhasselt, Filip Van Nieuwerburgh, Volkhard Kaever, Aurélie Crabbé, Tom Coenye

**Affiliations:** ^1^Laboratory of Pharmaceutical Microbiology, Ghent University, Ghent, Belgium; ^2^Department of Medical Microbiology, Ghent University Hospital, Ghent, Belgium; ^3^Laboratory of Pharmaceutical Biotechnology, Ghent University, Ghent, Belgium; ^4^Research Core Unit Metabolomics, Institute of Pharmacology, Hannover Medical School, Hanover, Germany

**Keywords:** coumarin, *Pseudomonas aeruginosa*, biofilm, virulence, type III secretion, c-di-GMP

## Abstract

As one of the major pathogens in wound infections, *Pseudomonas aeruginosa* produces several virulence factors and forms biofilms; these processes are under the regulation of various quorum sensing (QS) systems. Therefore, QS has been regarded as a promising target to treat *P. aeruginosa* infections. In the present study, we evaluated the effect of the plant-derived QS inhibitor coumarin on *P. aeruginosa* biofilms and virulence. Coumarin inhibited QS in the *P. aeruginosa* QSIS2 biosensor strain, reduced protease and pyocyanin production, and inhibited biofilm formation in microtiter plates in different *P. aeruginosa* strains. The effects of coumarin in inhibiting biofilm formation in an *in vitro* wound model and reducing *P. aeruginosa* virulence in the *Lucilia sericata* infection model were strain-dependent. Transcriptome analysis revealed that several key genes involved in the *las, rhl, Pseudomonas* quinolone signal (PQS), and integrated QS (IQS) systems were downregulated in coumarin-treated biofilms of *P. aeruginosa* PAO1. Coumarin also changed the expression of genes related to type III secretion and cyclic diguanylate (c-di-GMP) metabolism. The cellular c-di-GMP level of *P. aeruginosa* PAO1 and recent clinical *P. aeruginosa* strains was significantly reduced by coumarin. These results provide new evidence for the possible application of coumarin as an anti-biofilm and anti-virulence agent against *P. aeruginosa* in wound infections.

## Introduction

The opportunistic pathogen *Pseudomonas aeruginosa* frequently causes diverse infections in immunocompromised patients (Lyczak et al., 2000; Obritsch et al., 2005; Gellatly and Hancock, 2013), and is involved in both acute and chronic wound infections associated with high morbidity and mortality. Chronic wounds such as diabetic ulcers, venous ulcers, and pressure ulcers affect millions of patients worldwide and lead to high costs for the healthcare system (e.g., they represent an estimated cost of around 25 billion per year in the United States alone) (Sen et al., 2009). Infections in burn wounds also pose a heavy medical and economic burden in both developed and developing countries (McManus et al., 1985; Holder, 1993). Wound infections with *P. aeruginosa* are especially difficult to treat and are often associated with worse outcomes compared to other pathogens (Ünal et al., 2005), due to the extensive arsenal of virulence factors and increasing antibiotic resistance (Hirsch and Tam, 2010; Strateva and Mitov, 2011). In addition, biofilms formed by *P. aeruginosa* in wound infections further protect the bacteria from host immune defense and antimicrobials, impeding the healing process and triggering the shift to chronic wounds (Rybtke et al., 2011; Mulcahy et al., 2014). Therefore, there is an urgent need to develop alternative strategies to combat biofilm-related *P. aeruginosa* infections.

Quorum sensing (QS) is the intercellular communication process based on the production and detection of, and group-level response to, signal molecules (Waters and Bassler, 2005). The complex *P. aeruginosa* QS network has intensively been studied in the past decades as QS plays a crucial role in coordinating the production of several important virulence factors, including pyocyanin, protease, exotoxin A, hydrogen cyanide, and rhamnolipid (Smith and Iglewski, 2003). QS also affects biofilm formation and antibiotic resistance through multiple distinct mechanisms (Shih and Huang, 2002; Bjarnsholt et al., 2005; De Kievit, 2009; Rasamiravaka and El Jaziri, 2016). So far, four interacting QS systems have been identified in *P. aeruginosa*, including the *N*-acyl-homoserine lactone (AHL)-based *rhl* and *las* systems, the *Pseudomonas* quinolone signal (PQS) system, and the recently identified integrated QS (IQS) system (Lee and Zhang, 2015). This QS network allows *P. aeruginosa* to secrete extracellular virulence factors only when they can be produced at a sufficiently high level to overcome the host defense (Van Delden and Iglewski, 1998). In addition, QS has been reported to be involved in the spread of *P. aeruginosa* in burn wound infections (Rumbaugh et al., 1999).

Quorum sensing inhibition has been proposed as a promising anti-virulence strategy which would allow to “disarm” pathogens rather than killing them, and many potential QS inhibitors (QSIs) have been described (Kalia, 2013; LaSarre and Federle, 2013; Brackman and Coenye, 2015). A wide range of structurally different QSIs targeting *P. aeruginosa* have been identified, both from natural and synthetic sources (Jakobsen et al., 2013). The first comprehensively studied QSI is the furanone compound C-30 (Hentzer et al., 2003), which increased *P. aeruginosa* biofilm susceptibility to tobramycin and led to more efficient clearance of bacteria in a pulmonary mouse infection model (Wu et al., 2004). Ajoene, a sulfur-rich molecule from garlic, reduces expression of several QS-regulated virulence factors by activating the QS negative regulator RsmA through two small regulatory RNAs, RsmY, and RsmZ (Jakobsen et al., 2012, 2017). Many other QSIs such as 6-gingerol (Kim et al., 2015) and quercetin (Ouyang et al., 2016) have also been reported to reduce the virulence and biofilm formation of *P. aeruginosa*. These studies together demonstrate the possibility of using QSIs to control *P. aeruginosa* infections *in vitro* and/or in animal infection models.

Coumarin is a plant-derived phenolic compound and its derivatives are known for their anti-tumor and anti-inflammatory activities (Fylaktakidou et al., 2004; Kim et al., 2015; Reen et al., 2018). Coumarin has been described as an inhibitor of QS in *P. aeruginosa* and several other gram-negative bacteria (Gutiérrez-Barranquero et al., 2015). It was shown to inhibit biofilm formation, phenazine production, and motility in *P. aeruginosa* strain PA14 (Gutiérrez-Barranquero et al., 2015) and suppress virulence in *Vibrio splendidus* (Zhang et al., 2017). However, the mechanism by which coumarin inhibits QS has not been elucidated yet.

The goal of the present study was to evaluate the potential role of coumarin in the treatment of *P. aeruginosa*-infected wounds. To this end, we determined the effect of coumarin on the production of virulence factors and biofilm formation in an *in vitro* wound model, using the *P. aeruginosa* reference isolate PAO1 as well as several *P. aeruginosa* clinical wound isolates. We also investigated the effect of coumarin on *P. aeruginosa* virulence to *Lucilia sericata* maggots, which are widely used in the debridement of chronic wounds (Chambers et al., 2003). The QS-regulated virulence factors of *P. aeruginosa* are responsible for killing of these maggots and often lead to the failure of maggot therapy in wounds infected with *P. aeruginosa* (Andersen et al., 2010). Therefore, we explored the possible use of coumarin as a pre-treatment to inhibit the QS controlled virulence of *P. aeruginosa* and increase the survival of maggots when treating wound infections heavily colonized with *P. aeruginosa*. Finally, transcriptome analysis in both planktonic and biofilm cells was performed to identify genes involved in the inhibition of QS by coumarin.

## Materials and Methods

### Bacterial Strains, Chemicals, and Growth Media

*Pseudomonas aeruginosa* strains 1803, 2063, 2091, 2549, and 3120 were isolated from wound infections in 2017 and obtained from the Ghent University Hospital, Belgium. Reference strain *P. aeruginosa* PAO1 and these five wound isolates were routinely cultured in Luria-Bertani (LB) agar or broth (Lab M limited, United Kingdom) or Tryptic Soy Agar (TSA, Lab M limited, United Kingdom). The QS inhibition selector *P. aeruginosa* QSIS2 (Rasmussen et al., 2005) was cultured in ABT minimal medium (AB medium, containing 2.5 mg/L thiamine) supplemented with 0.5% glucose, 0.5% casamino acids, and 80 μg/mL gentamicin. All strains were grown aerobically at 37°C. Coumarin was purchased from Sigma-Aldrich (Bornem, Belgium) and dissolved in dimethyl sulfoxide (DMSO) (Alfa Aesar, Germany) as stock solutions (1 M). *L. sericata* maggots in BioBags were purchased from BioMonde (Germany), and were used immediately after receipt.

### QS Inhibition Assay

The *P. aeruginosa* QSIS2 reporter strain is a *lasI rhlI* double mutant containing plasmid pLasB-SacB1 encoding an AHL-induced killing system (Rasmussen et al., 2005). The QS inhibition assay based on QSIS2 was performed as previously described, with minor modifications (Brackman et al., 2009). Briefly, an overnight culture of QSIS2 was diluted in ABT medium to an optical density at 590 nm (OD590) of 0.1. Cell suspensions (50 μL) and 50 μL LB with sucrose (224 mg/mL) was added to 96-well microtiter plates, supplemented with gentamicin at a final concentration of 80 μg/mL. *N*-(3-oxododecanoyl)-L-homoserine lactone (3-oxo-C12-HSL) and *N*-butyryl-L-homoserine lactone (C4-HSL) (200 nM each, Sigma) were added to activate the QS system as positive controls, and sterile MilliQ ultrapure water was used as a negative control. To test the QS inhibition effect, coumarin was added in a final concentration of 1, 1.5, 2, 2.5, and 3 mM. The microtiter plates were incubated for 10 h at 37°C, and the absorbance was measured at 450 nm using an EnVision Multilabel Reader (Perkin Elmer, Waltham, MA, United States). The difference in growth between the negative control without AHLs and the positive control with AHLs was considered as 100% QS inhibition. Three independent experiments were performed with three replicates each (*n* = 9).

### Measurements of Pyocyanin and Protease Production

Overnight cultures of *P. aeruginosa* strains were standardized to OD590 0.5 and 1:50 diluted in 10 mL LB with or without coumarin (2 mM). After 24 h incubation at 37°C, the supernatant was collected and filter-sterilized. For the pyocyanin assay, chloroform (3 mL) was added to 5 mL *P. aeruginosa* culture supernatant and vortexed. The chloroform phase was kept after centrifugation (5000 rpm, 5 min), and mixed with 1 mL HCl (0.2 M). The absorbance of the pink HCl layer was measured at 520 nm with the EnVision Multilabel Reader after vortexing and centrifugation (Krishnan et al., 2012). 0.2 M HCl was used as a negative control in the measurement of absorbance. Protease production was tested by the azocasein assay as described previously (Nicodeme et al., 2005; Vandecandelaere et al., 2014) with modifications. Azocasein (Sigma) solution (400 μL, 5 mg/mL 0.1 M Tris-HCl buffer) was mixed with 400 μL supernatant and incubated at 37°C for 1 h. The reaction was stopped by adding 100 μL 10% (w/v) trichloroacetic acid and the mixture was then centrifuged. 100 μL of the resulting supernatant was transferred to 96-well microtiter plates and mixed with 100 μL 625 mM NaOH. The absorbance was measured at 420 nm with an EnVision Multilabel Reader. Azocasein solution with LB medium was processed following the same procedure and used as blank control. Two independent experiments were performed with three replicates each (*n* = 6).

### The Effect of Coumarin on Planktonic Cell Growth and on Biofilm Formation in Microtiter Plates

Overnight cultures of *P. aeruginosa* strains were standardized to OD590 0.5 and inoculated in 200 μL of LB with 1:50 dilutions with or without coumarin in microtiter plates at 37°C. The growth of planktonic cells was quantified by measuring the absorbance at 450 nm (in order to avoid the interference of pyocyanin) using an EnVision Multilabel Reader (Perkin Elmer, Waltham, MA, United States). Three independent experiments were performed (*n* = 3).

Biofilm formation in microtiter plates was evaluated as described previously (Brackman et al., 2009). Briefly, overnight cultures of *P. aeruginosa* strains were diluted to approximately 5 × 10^7^ CFU/mL in LB broth. 100 μL of the suspension was transferred to the wells of a round-bottomed 96-well microtiter plate with coumarin in a final concentration of 1 mM or 2 mM. 10 μL MilliQ with DMSO was added for the control. The plate was incubated at 37°C for 4 h, and then the wells were rinsed once with sterile physiological saline (PS) and re-filled with fresh medium and coumarin. The plate was incubated at 37°C for an additional 20 h. After removing the supernatant and washing the wells once with sterile PS, two cycles of vortexing (5 min) and sonication (5 min) were performed to release biofilm cells, and the number of CFU/biofilm was determined by plating. Three independent experiments were performed with three replicates each (*n* = 9).

### The Effect of Coumarin on Biofilm Formation in a Wound Model

The biofilm wound model used has been described before (Brackman et al., 2016). A spongy artificial dermis of 1 cm^3^ (upper layer: chemically cross-linked hyaluronic acid, lower layer: hyaluronic acid and collagen) is used as a substrate for biofilm formation to mimic biofilm formation at the air-liquid interface in real wounds. Each sheet of artificial dermis was placed in a 24-well microtiter plate. 300 μL medium containing Bolton Broth, heparinized bovine plasma and freeze-thaw laked horse blood was added on dermis. Coumarin was added in a final concentration of 2 mM. *P. aeruginosa* suspensions (10 μL) containing approximately 10^4^ bacterial cells were added on top of the dermis. After 24 h, the infected dermis was washed with 1 mL PS and was transferred into 9 mL PS. Biofilm cells on the dermis were collected by three cycles of vortexing (30 s) and sonication (30 s). The number of CFU/dermis was quantified by plating. Three independent experiments were performed with three replicates each (*n* = 9).

### The Effect of Coumarin on *P. aeruginosa* Virulence to *L. sericata*

Blood agar (5% horse blood in LB agar) was prepared in 6 cm Petri dishes with DMSO as control or with coumarin (2 mM in a final concentration). *P. aeruginosa* bacteria (5 × 10^5^ CFU) were spread on the blood agar and incubated overnight at 37°C before the assay. The maggots were aseptically transferred onto the blood agar (10 maggots per plate) and cultured at 37°C in the dark (*n* = 50 in total from two independent experiments). Maggot survival was assessed after 24 h. Immobile/inactive maggots were considered to be dead, and maggot death was verified by stimulating the maggots with an inoculation needle.

### RNA Extraction, Sequencing and Analysis

RNA sequencing was performed on coumarin-treated (2 mM, 24 h) and untreated *P. aeruginosa* PAO1 biofilms. To detect the initial changes on gene expression due to the coumarin treatment also shortly treated (2 mM, 1 h) and untreated planktonic cultures were included. Suspensions of planktonic cells (5 × 10^7^ CFU/mL) were added to a 96-well microtiter plate and treated with 2 mM coumarin or DMSO as control. After incubation at 37°C for 1 h, cells were collected on ice for RNA extraction. Biofilms were formed in microtiter plates with 2 mM coumarin or DMSO and cells from 24 h-biofilms were collected as described above. Subsequently, total RNA was extracted immediately using the Ambion RiboPure^TM^ RNA Purification Kit (Life Technologies, Renfrewshire, United Kingdom) according to the manufacturer’s instructions. Three biological replicates were performed for each condition.

RNA sequencing was performed as described previously (Van Acker et al., 2014). Total RNA of each sample was depleted for ribosomal RNA using the Ribo-Zero Magnetic Kit for gram-negative bacteria (Epicentre, Madison, WI, United States). Truseq stranded RNA library preparation kit (Illumina) was then used to create strand-specific cDNA sequencing libraries. Quality control of the libraries (DNA 1000 chip, Agilent Technologies, Santa Clara, CA, United States) was performed and the concentration was determined according to recommendations provided by Illumina. Sequencing was performed using an Illumina NextSeq 500, generating 75 bp unpaired reads. Fastq files were deposited at ArrayExpress under the accession number E-MTAB-6629. Using CLC Biosystems Genomic Workbench 10.1.1 (CLC Bio, Qiagen, Waltham, MA, United States), quality trimming was performed and the quality filtered reads of each sample were mapped (length fraction 0.8 and similarity fraction 0.8) against the genome sequence of *P. aeruginosa* PAO1 (the NCBI reference sequence with accession number NC_002516). Total gene read values are used by the differential expression for RNA-Seq tool based on a negative binomial generalized linear model (GLM) in CLC Workbench. Only genes that were significantly differentially regulated (the false discovery rate adjusted *p*-value < 0.05) and with at least 1.5-fold change compared to the control were considered. Gene descriptions were obtained from the *Pseudomonas* Genome Database (Winsor et al., 2015).

### C-di-GMP Quantification

Quantifications of cyclic diguanylate (c-di-GMP) levels in *P. aeruginosa* cells were performed as described previously (Burhenne and Kaever, 2013). Briefly, an overnight culture of *P. aeruginosa* strains was standardized to OD590 0.5 and 200 μL was inoculated in 9.8 mL of LB with or without coumarin. After 24 h, the cells were harvested by centrifugation at 4°C for 20 min from 5 mL culture suspensions. The wet weight of collected cells was measured. Cells were lysed in ice-cold extraction buffer consisting of acetonitrile/methanol/water (2/2/1, v/v/v), and incubated at 4°C for 15 min. The cell suspension was then heated to 95°C for 10 min and centrifuged. The extraction of the resulting pellet was repeated twice with 200 μL of extraction solvent at 4°C omitting the heating step. The solvent of the combined supernatants was evaporated to dryness in a vacuum centrifuge. The pellets were dissolved in HPLC-grade water for analysis by liquid chromatography-coupled tandem mass spectrometry as described before (Burhenne and Kaever, 2013). Three independent experiments were performed (*n* = 3).

### Statistical Analyses

Numerical data were analyzed using GraphPad Prism 6.0 and presented as mean ± standard deviation (SD). The normal distribution of the data was checked by the D’Agostino-Pearson normality test. Normally distributed data were analyzed by one-way ANOVA or Student’s *t*-test, and non-normally distributed data were analyzed by the Kruskal–Wallis test or the Mann–Whitney test.

## Results

### Coumarin Inhibits QS in *P. aeruginosa* QSIS2 Biosensor

**FIGURE 1:**
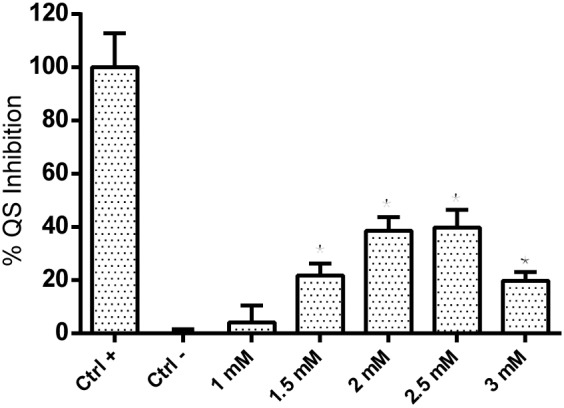
QS inhibition effect of coumarin detected by *P. aeruginosa* QSIS2 reporter strain. Data shown are the mean (*n* = 9), error bars represent standard deviation.^∗^*p* < 0.05 compared to negative control in one-way ANOVA.

The previously described QS inhibitory activity of coumarin was confirmed using the *P. aeruginosa* QSIS2 reporter strain, in which the QS inhibition effect was represented by the cell growth (A450). As shown in **Figure 1**, while 1 mM coumarin showed no significant QS inhibition effect, approx. 20∼40% QS inhibition could clearly be observed at concentrations of 1.5, 2, 2.5, and 3 mM compared with the negative control in which the QS system was fully activated.

### Coumarin Reduces Pyocyanin and Protease Production in Different *P. aeruginosa* Strains

**FIGURE 2:**
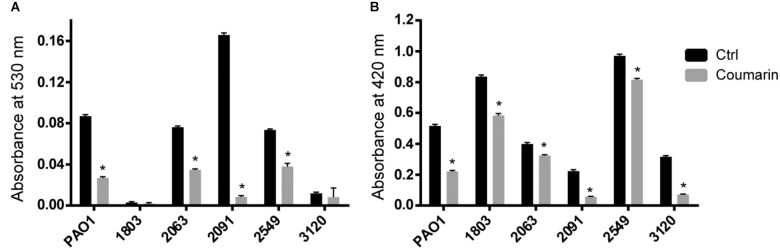
Effect of coumarin (2 mM) on pyocyanin **(A)** and protease **(B)** production in different *P. aeruginosa* strains. Data shown are the mean (*n* = 6), error bars represent standard deviation. ^∗^*p* < 0.05 compared to the untreated control group in Mann–Whitney test.

Pyocyanin production was significantly decreased by coumarin in strains PAO1, 2063, 2091, and 2549 (*p* < 0.05), but not in strains 1083 and 3120 which produced virtually no pyocyanin in the experimental conditions used (**Figure 2A**). Coumarin also reduced protease production in *P. aeruginosa* PAO1 and the five clinical strains, to varying degrees (*p* < 0.05) (**Figure 2B**). These results indicate that coumarin inhibits the production of QS-regulated virulence factors in different *P. aeruginosa* clinical strains.

### The Effect of Coumarin on *P. aeruginosa* Biofilms

**FIGURE 3:**
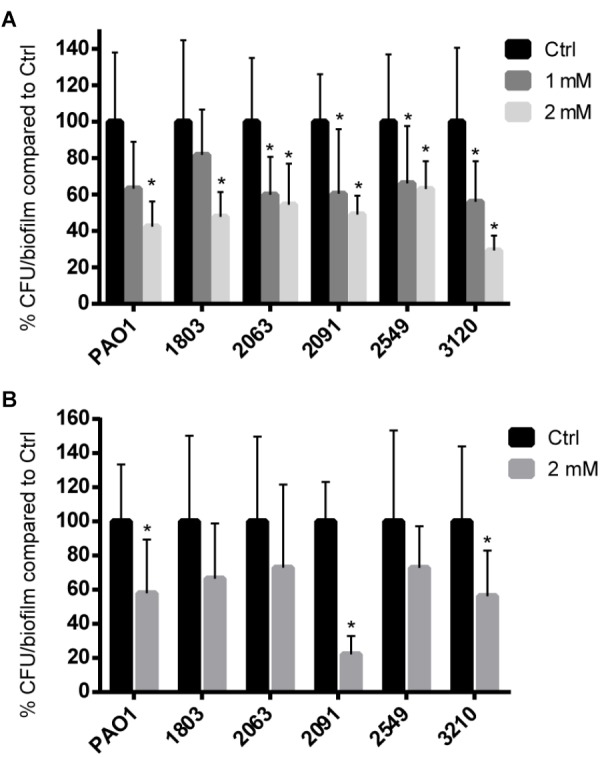
The effect of coumarin on *P. aeruginosa* biofilms. Data shown are the mean (*n* = 9), error bars represent standard deviation. **(A)** Biofilms formed in microtiter plates. ^∗^*p* < 0.05 compared to the untreated control group in one-way ANOVA or Kruskal–Wallis test. **(B)** Wound model biofilms treated with 2 mM coumarin. ^∗^*p* < 0.05 compared to the untreated control group in Student’s *t*-test or Mann–Whitney test.

No significant effect on growth of planktonic cells of *P. aeruginosa* strains was observed when 1 mM or 2 mM coumarin was added (**Supplementary Figure S1**). The effect of coumarin on biofilm formation of *P. aeruginosa* strains was tested in 96-well microtiter plates as well as in an *in vitro* wound model. In microtiter plates, biofilm formation of all *P. aeruginosa* strains investigated was significantly reduced in the presence of 2 mM coumarin (*p* < 0.05) (**Figure 3A**); for four strains (2063, 2091, 2549, and 3120) this was also the case in the presence of 1 mM coumarin.

In the wound model, statistically significant reduced biofilm formation in the presence of 2 mM coumarin was observed for *P. aeruginosa* PAO1, 2091, and 3210 (*p* < 0.05) (**Figure 3B**). Biofilm formation for the other three clinical strains showed a non-significant decrease of approx. 30%.

### The Effect of Coumarin on *P. aeruginosa* Virulence to *L. sericata*

**FIGURE 4:**
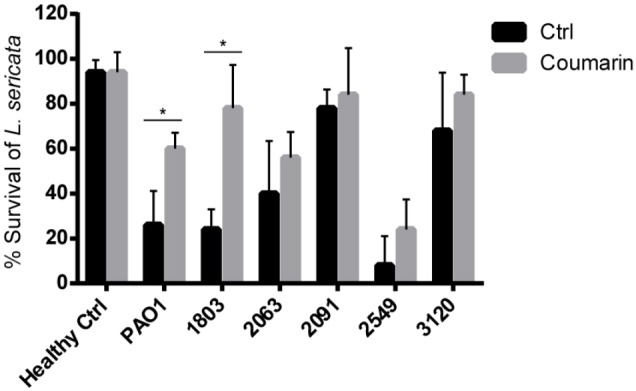
The effect of coumarin (2 mM) on survival of *L. sericata*. Data shown are the mean (*n* = 9), error bars represent standard deviation. ^∗^*p* < 0.05 compared to the untreated control group in Mann–Whitney test.

As shown in **Figure 4**, the virulence of *P. aeruginosa* to *L. sericata* varied between different clinical isolates. *P. aeruginosa* 2549 appeared to be most virulent among the strains tested, as exposure of the larvae to this strain led to >90% killing after 24 h. In the coumarin-treated groups, significant increase in *L. sericata* survival was observed for PAO1 and 1803, with 34% and 54% more survival compared to the control groups, respectively. For the other *P. aeruginosa* strains, coumarin increased the survival of *L. sericata* by 6∼16% but this was not significant. It should, however, be noted that for two of these strains (2091 and 3120) survival already was quite high in the control group, suggesting these strains are less virulent in this model.

### Transcriptome Analysis of *P. aeruginosa* Planktonic and Biofilm Cells Treated With Coumarin

In order to obtain insight into the mechanism(s) behind the QS inhibitory effect of coumarin, RNA sequencing was performed on *P. aeruginosa* PAO1 planktonic cells after 1 h coumarin treatment (2 mM) and biofilm cells grown in the presence of coumarin (2 mM) for 24 h. In planktonic cells, 46 genes were downregulated and 21 genes were upregulated by coumarin (**Supplementary Table S1**). Nearly half of the downregulated genes (21) were related to the type III secretion system (T3SS), including the key genes coding for the needle complex (*pscF, pscJ*, and *pscL*), the translocation apparatus (*pcrV, popB*, and *popD*), the chaperones (*pcrH* and *pscG*), as well as the effector proteins (*exoS* and *exoT*). Two sets of two-component regulator systems were downregulated (PA4774/PA4775 and *pmrAB*). An oxidative stress sensing regulator *ospR* was repressed, as well as gene PA2826 (encoding a glutathione peroxidase which is under the regulation of *ospR*) (Lan et al., 2010). This result indicated that coumarin might affect the response of *P. aeruginosa* to oxidative stress. One HD-GYP domain phosphodiesterase (PDE) encoding gene (PA4781) was downregulated; PA4781 has been reported to degrade c-di-GMP (Ryan et al., 2009; Stelitano et al., 2013; Rinaldo et al., 2015). However, no significant changes in either QS or QS-regulated virulence genes were observed in coumarin-treated planktonic samples, except that *phzG2* involved in phenazine production was repressed by over 50-fold.

In coumarin-treated biofilms, 399 genes were significantly downregulated (**Supplementary Table S2**). Among them, several core genes involved in the four different QS systems of *P. aeruginosa* were repressed. The AHL synthase encoding genes *lasI* and *rhlI* were both downregulated, as was the receptor encoding gene *rhlR*. The expression of *lasR* was not significantly influenced. Gene *pqsB* and *pqsC* within the *pqsABCDE* operon (which controls PQS synthesis) were both downregulated, as was *pqsH* (involved in the conversion of 2-heptyl-4-quinolone to PQS). No difference was observed in the expression of the gene encoding the PQS receptor PqsR. Moreover, genes responsible for the synthesis of IQS (*ambBCDE*) were also significantly downregulated in coumarin-treated biofilms. In addition, genes involved in the production of QS-regulated virulence factors including alkaline protease, hydrogen cyanide, LasA protease, LasB elastase, phenazine, siderophores, and rhamnolipids were significantly downregulated. These results confirm that coumarin interferes with the QS network of *P. aeruginosa* and the production of QS-related virulence factors. Genes involved in T3SS were repressed by coumarin in the biofilm cells (**Supplementary Table S2**), including several genes encoding T3SS regulators (*exsC, exsD*, and *ptrA*). The c-di-GMP PDE encoding gene PA4781 was downregulated in coumarin-treated biofilm cells by 2-fold.

In addition, 234 genes were upregulated in the coumarin-treated biofilm cells (**Supplementary Table S3**). PA2226 encoding a negative QS regulator QsrO was upregulated by 3.7-fold. Overexpression of QsrO has been reported to repress the *las, rhl*, and PQS systems of *P. aeruginosa*, although the detailed mechanisms have not been elucidated yet (Köhler et al., 2014). Co-expression of PA2226 and PA2225 prevents the induction of T3SS (Köhler et al., 2014), and PA2225 was found to be upregulated by 2.0-fold in the coumarin-treated biofilm. *tpbA*, encoding a tyrosine phosphatase that represses c-di-GMP production (Ueda and Wood, 2009) was upregulated by 4.0-fold in the coumarin-treated biofilm cells. No significant changes in the expression of other diguanylate cyclase (DGC) or PDE genes involved in c-di-GMP metabolism were found in coumarin-treated biofilm cells.

### Coumarin Reduced C-di-GMP Level in *P. aeruginosa*

**FIGURE 5:**
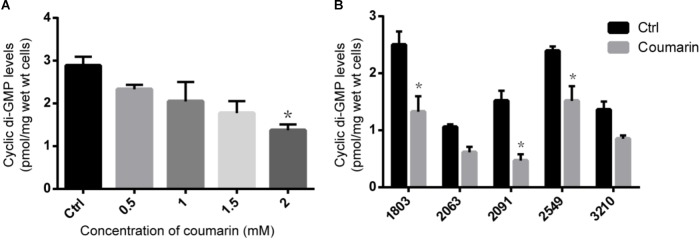
The effect of coumarin on c-di-GMP level in *P. aeruginosa*. **(A)** C-di-GMP level of *P. aeruginosa* PAO1 treated with different concentrations of coumarin. **(B)** C-di-GMP level in different *P. aeruginosa* clinical strains treated with 2 mM coumarin. ^∗^*p* < 0.05 compared to the untreated control in one-way ANOVA or Kruskal–Wallis test. Data shown are the mean (*n* = 3), error bars represent standard deviation.

To confirm the influence of coumarin on c-di-GMP metabolism, we determined intracellular c-di-GMP concentrations of planktonic *P. aeruginosa* cells. As shown in **Figure 5A**, the c-di-GMP concentration in the untreated PAO1 control was 2.89 ± 0.21 pmol/mg of wet cells, while the intracellular c-di-GMP level in PAO1 treated with 2 mM coumarin was significantly reduced (*p* < 0.05) to 1.37 ± 0.13 pmol/mg of wet cells. When treated with 2 mM coumarin, the c-di-GMP concentration in the five clinical *P. aeruginosa* strains investigated, was reduced by 36.4∼69.7% compared to the untreated control (**Figure 5B**).

## Discussion

Coumarin was previously described as a QSI in *P. aeruginosa* (Gutiérrez-Barranquero et al., 2015) and this was confirmed in the present study. In addition, coumarin reduced protease and pyocyanin production in different *P. aeruginosa* strains, as well as biofilm formation both in microtiter plates and in an *in vitro* wound model. We also demonstrated that coumarin increases the survival of *L. sericata* maggots in the presence of *P. aeruginosa* PAO1 and recent clinical strains. A transcriptomic analysis demonstrated that the expression of a large number of genes (including genes involved in QS and T3SS) is regulated in response to coumarin in the biofilms of *P. aeruginosa*, and intracellular c-di-GMP levels were lower in treated cells, compared to untreated controls.

Several studies have shown that clinical *P. aeruginosa* strains display varying levels of virulence, and highlighted the importance to include clinical isolates when evaluating the effect of potential novel treatment approaches (García-Contreras et al., 2015; Guendouze et al., 2017). We observed that different *P. aeruginosa* clinical isolates indeed vary significantly in their ability to produce protease and pyocyanin, as well as in their virulence towards *L. sericata*. Coumarin significantly inhibited biofilm formation in microtiter plates for all *P. aeruginosa* strains tested, whereas its biofilm-inhibitory effect in the wound model and its effect on virulence in *L. sericata* were strain-dependent, suggesting that coumarin’s effect as QSI is reduced in the more complex *in vivo* environment and that the therapeutic role of coumarin in treating wound infections and helping with maggot-based debridement therapy may be limited. The strain-dependent effects in clinical *P. aeruginosa* isolates have also been reported for other QS inhibition agents, such as furanone C-30 (García-Contreras et al., 2015), 5-fluorouracil (García-Contreras et al., 2013), ZnO nanoparticles (García-Lara et al., 2015), and the quorum quenching enzyme SsoPox (Guendouze et al., 2017). Combined, these data indicate that in many cases QSI are less effective in clinical strains and whether these QSI can be developed into effective anti-virulence drugs remains to be determined (which will require more data in animal models and on a larger number of clinical strains).

To obtain a global picture of effect of coumarin on QS, biofilm, and virulence of *P. aeruginosa*, we performed transcriptome analysis on *P. aeruginosa* PAO1 planktonic cells after 1 h of treatment with coumarin, as well as on biofilms treated with coumarin for 24 h. The expression of genes involved in the QS system of *P. aeruginosa* was not significantly changed in planktonic cells after 1 h. In a previous study, coumarin (1.36 mM) has been reported to reduce the expression of *pqsA* and *rhlI* in planktonic cells after 6 h and 24 h, respectively, while *lasI* expression was not affected either at 6 h or 24 h (Gutiérrez-Barranquero et al., 2015). In biofilm cells we found that *lasI* expression was downregulated by coumarin, as well as *rhlI, rhlR*, and genes within the *pqsABCDE* and *ambBCDE* operons. These results confirm that coumarin interferes with the *P. aeruginosa* QS network. The observation that the expression of QS-regulated virulence genes was downregulated in *P. aeruginosa* PAO1 was in line with the decreased protease and pyocyanin production and reduced virulence observed in *L. sericata*. Also *qsrO*, encoding a negative regulator of QS was overexpressed in coumarin-treated biofilms suggesting that this regulator contributes to the inhibition of QS by coumarin.

T3SS acts as a major virulence determinant in *P. aeruginosa* that manipulates host cell responses and plays an important role in acute infections (Hauser, 2009). A microarray-based analysis of *P. aeruginosa* QS regulons revealed that at least three genes involved in T3SS (*pscQ, pscI*, and *pcrH*) are negatively regulated by QS (Wagner et al., 2003). Bleves et al. (2005) have also reported that the expression of T3SS in *P. aeruginosa* is negatively regulated by QS, especially by RhlR under low calcium levels. These results raise the concern that interfering with QS might potentially increase the T3SS-related virulence in *P. aeruginosa* (García-Contreras, 2016). However, transcriptome data obtained from *P. aeruginosa* treated with coumarin in the present study and 6-gingerol, another QSI (Kim et al., 2015) revealed that genes involved in T3SS were repressed. These results suggest that although QS has been reported to negatively affect T3SS expression, QS inhibition by certain QSIs may not necessarily induce T3SS-related virulence. In contrast, it is possible to inhibit QS and T3SS simultaneously by QSIs such as coumarin and 6-gingerol, although the underlying mechanisms remain to be elucidated.

C-di-GMP regulates many bacterial behaviors, and a high intracellular c-di-GMP concentration has been reported to promote biofilm formation in *P. aeruginosa* and other bacteria (Römling et al., 2013). The intracellular concentration of c-di-GMP is determined by GGDEF domain-containing DGCs (which synthesize c-di-GMP), and EAL or HD-GYP domain-containing PDEs (which degrade c-di-GMP) (Römling et al., 2013). We found that coumarin affected two genes involved in c-di-GMP metabolism in *P. aeruginosa* PAO1, i.e., PA4781 and *tpbA*, and demonstrated that coumarin significantly reduced the c-di-GMP level in *P. aeruginosa*. We hypothesize this is due to the upregulation of *tpbA*. TpbA acts as a negative regulator of c-di-GMP production, which deactivates the GGDEF-domain DGC TpbB responsible for c-di-GMP synthesis and indirectly reduces c-di-GMP levels in *P. aeruginosa* (Ueda and Wood, 2009). This effect is apparently stronger than the consequence of downregulation of PA4781, what on itself would result in increased c-di-GMP levels.

Our results indicate that coumarin not only acts as a QSI but also reduces c-di-GMP levels, and these combined effects may explain the reduced biofilm formation observed. The connections between QS and c-di-GMP in *P. aeruginosa* have just begun to be elucidated. Mutants of *P. aeruginosa* PA14 without functional *lasI, lasR*, or *rhlR* showed decreased expression of *tpbA* (Ueda and Wood, 2009), leading to the assumption that QS negatively regulates c-di-GMP levels through TpbA/TpbB. However, our results showed that coumarin inhibits QS-related genes, but the expression of *tpbA* was significantly upregulated by coumarin and led to reduced c-di-GMP level. Another study showed that *rhl* and PQS systems were expressed at a higher level in *P. aeruginosa* PAO1 with reduced c-di-GMP level due to overexpression of *yhjH* (encoding a PDE) than in a *wspF* mutant with elevated c-di-GMP levels (Lin Chua et al., 2017). This suggested that low c-di-GMP levels could increase expression of QS-related genes and the production of QS-regulated virulence factors. In contrast, our results showed that coumarin reduced both c-di-GMP levels and QS-regulated virulence. These results might be explained by the different mechanisms leading to the reduced c-di-GMP level. The distinct gene expression profiles (431 upregulated genes and 595 downregulated genes) observed in the study of Lin Chua et al. is directly due to the loss of *wspF* and overexpression of *yhjH*, whereas in our study no change was observed in *wspF* expression.

Several other compounds of the coumarin class have also been reported for their anti-QS and anti-biofilm effects, e.g., esculetin, esculin, and umbelliferone (Zeng et al., 2008; Lee et al., 2014). Our research on the prototype coumarin molecule in this family revealed that coumarin can inhibit not only QS but also T3SS and c-di-GMP signaling, leading to reduced virulence and biofilm formation in *P. aeruginosa*. These results increase our understanding of the molecular mechanism(s) involved in the activity of coumarin and related molecules, and suggest these molecules could be useful to combat biofilm-related infections. Although the effects of coumarin in inhibiting *P. aeruginosa* biofilm formation in a wound model and virulence to *L. sericata* were found to be strain-dependent, structural modifications based on the coumarin scaffold may allow the development of more active coumarin-derivatives with potential application in the treatment of *P. aeruginosa* infections.

## Conclusion

• Coumarin can reduce QS-regulated virulence and biofilm formation in *P. aeruginosa* strains, but its effect on *P. aeruginosa* biofilm formed in wound model and the virulence to *L. sericata* maggots is strain-dependent.
• Coumarin downregulates the expression of key genes involved in the *las, rhl*, PQS, and IQS systems in the biofilm of *P. aeruginosa*.
• Coumarin reduces the expression of genes related to type III secretion, and decreases the cellular c-di-GMP level in *P. aeruginosa*.

## Author Contributions

TC, YZ, AS, and AC conceived and designed the experiments. YZ performed the experiments. AS, HVA, and FVN helped with RNA-sequencing and data analysis. JW and VK contributed to c-di-GMP quantification. All authors contributed to the writing.

## Conflict of Interest Statement

The authors declare that the research was conducted in the absence of any commercial or financial relationships that could be construed as a potential conflict of interest.

## References

[B1] Andersen A. Joergensen B. Bjarnsholt T. Johansen H. Karlsmark T. Givskov M. (2010). Quorum-sensing-regulated virulence factors in *Pseudomonas aeruginosa* are toxic to *Lucilia sericata* maggots. *Microbiology* 156 400–407. 10.1099/mic.0.032730-0 19892758 PMC2885677

[B2] Bjarnsholt T. Jensen P. Ø Burmølle M. Hentzer M. Haagensen J. A. Hougen H. P. (2005). *Pseudomonas aeruginosa* tolerance to tobramycin, hydrogen peroxide and polymorphonuclear leukocytes is quorum-sensing dependent. *Microbiology* 151 373–383. 10.1099/mic.0.27463-0 15699188

[B3] Bleves S. Soscia C. Nogueira-Orlandi P. Lazdunski A. Filloux A. (2005). Quorum sensing negatively controls type III secretion regulon expression in *Pseudomonas aeruginosa* PAO1. *J. Bacteriol.* 187 3898–3902. 10.1128/JB.187.11.3898-3902.2005 15901720 PMC1112058

[B4] Brackman G. Coenye T. (2015). Quorum sensing inhibitors as anti-biofilm agents. *Curr. Pharm. Des.* 21 5–11. 10.2174/1381612820666140905114627 25189863

[B5] Brackman G. Garcia-Fernandez M. J. Lenoir J. De Meyer L. Remon J. P. De Beer T. (2016). Dressings loaded with cyclodextrin–hamamelitannin complexes increase Staphylococcus aureus susceptibility toward antibiotics both in single as well as in mixed biofilm communities. *Macromol. Biosci.* 16 859–869. 10.1002/mabi.201500437 26891369

[B6] Brackman G. Hillaert U. Van Calenbergh S. Nelis H. J. Coenye T. (2009). Use of quorum sensing inhibitors to interfere with biofilm formation and development in *Burkholderia multivorans* and *Burkholderia cenocepacia*. *Res. Microbiol.* 160 144–151. 10.1016/j.resmic.2008.12.003 19146953

[B7] Burhenne H. Kaever V. (2013). *“Quantification of Cyclic Dinucleotides by Reversed-phase LC-MS/MS,” in Cyclic Nucleotide Signaling in Plants*. Totowa, NJ: Humana Press, 27–37. 10.1007/978-1-62703-441-8_3 23681570

[B8] Chambers L. Woodrow S. Brown A. Harris P. Phillips D. Hall M. (2003). Degradation of extracellular matrix components by defined proteinases from the greenbottle larva *Lucilia sericata* used for the clinical debridement of non-healing wounds. *Br. J. Dermatol.* 148 14–23. 10.1046/j.1365-2133.2003.04935.x 12534589

[B9] De Kievit T. (2009). Quorum sensing in *Pseudomonas aeruginosa* biofilms. *Environ. Microbiol.* 11 279–288. 10.1111/j.1462-2920.2008.01792.x 19196266

[B10] Fylaktakidou K. C. Hadjipavlou-Litina D. J. Litinas K. E. Nicolaides D. N. (2004). Natural and synthetic coumarin derivatives with anti-inflammatory/antioxidant activities. *Curr. Pharm. Des.* 10 3813–3833. 10.2174/1381612043382710 15579073

[B11] García-Contreras R. (2016). Is quorum sensing interference a viable alternative to treat *Pseudomonas aeruginosa* infections? *Front. Microbiol.* 7:1454. 10.3389/fmicb.2016.01454 27683577 PMC5021973

[B12] García-Contreras R. Martínez-Vázquez M. Velázquez Guadarrama N. Villegas Pañeda A. G. Hashimoto T. Maeda T. (2013). Resistance to the quorum-quenching compounds brominated furanone C-30 and 5-fluorouracil in *Pseudomonas aeruginosa* clinical isolates. *Pathog. Dis.* 68 8–11. 10.1111/2049-632X.12039 23620228

[B13] García-Contreras R. Peréz-Eretza B. Jasso-Chávez R. Lira-Silva E. Roldán-Sánchez J. A. González-Valdez A. (2015). High variability in quorum quenching and growth inhibition by furanone C-30 in *Pseudomonas aeruginosa* clinical isolates from cystic fibrosis patients. *Pathog. Dis.* 73:ftv040. 10.1093/femspd/ftv040 26048733

[B14] García-Lara B. Saucedo-Mora M. Roldán-Sánchez J. Pérez-Eretza B. Ramasamy M. Lee J. (2015). Inhibition of quorum-sensing-dependent virulence factors and biofilm formation of clinical and environmental *Pseudomonas aeruginosa* strains by ZnO nanoparticles. *Lett. Appl. Microbiol.* 61 299–305. 10.1111/lam.12456 26084709

[B15] Gellatly S. L. Hancock R. E. (2013). *Pseudomonas aeruginosa*: new insights into pathogenesis and host defenses. *Pathog. Dis.* 67 159–173. 10.1111/2049-632X.12033 23620179

[B16] Guendouze A. Plener L. Bzdrenga J. Jacquet P. Rémy B. Elias M. (2017). Effect of quorum quenching lactonase in clinical isolates of *Pseudomonas aeruginosa* and comparison with quorum sensing inhibitors. *Front. Microbiol.* 8:227. 10.3389/fmicb.2017.00227 28261183 PMC5306132

[B17] Gutiérrez-Barranquero J. A. Reen F. J. McCarthy R. R. O’Gara F. (2015). Deciphering the role of coumarin as a novel quorum sensing inhibitor suppressing virulence phenotypes in bacterial pathogens. *Appl. Microbiol. Biotechnol.* 99 3303–3316. 10.1007/s00253-015-6436-1 25672848

[B18] Hauser A. R. (2009). The type III secretion system of *Pseudomonas aeruginosa*: infection by injection. *Nat. Rev. Microbiol.* 7:654. 10.1038/nrmicro2199 19680249 PMC2766515

[B19] Hentzer M. Wu H. Andersen J. B. Riedel K. Rasmussen T. B. Bagge N. (2003). Attenuation of *Pseudomonas aeruginosa* virulence by quorum sensing inhibitors. *EMBO J.* 22 3803–3815. 10.1093/emboj/cdg366 12881415 PMC169039

[B20] Hirsch E. B. Tam V. H. (2010). Impact of multidrug-resistant *Pseudomonas aeruginosa* infection on patient outcomes. *Exp. Revi. Pharmacoecon. Outcomes Res.* 10 441–451. 10.1586/erp.10.49 20715920 PMC3071543

[B21] Holder I. A. (1993). *“P. aeruginosa Burn Infections: Pathogenesis and Treatment,” in Pseudomonas aeruginosa as an Opportunistic Pathogen*. Boston, MA: Springer, 275–295. 10.1007/978-1-4615-3036-7_14

[B22] Jakobsen T. H. Bjarnsholt T. Jensen P. Ø Givskov M. Høiby N. (2013). Targeting quorum sensing in *Pseudomonas aeruginosa* biofilms: current and emerging inhibitors. *Future Microbiol.* 8 901–921. 10.2217/fmb.13.57 23841636

[B23] Jakobsen T. H. van Gennip M. Phipps R. K. Shanmugham M. S. Christensen L. D. Alhede M. (2012). Ajoene, a sulfur-rich molecule from garlic, inhibits genes controlled by quorum sensing. *Antimicrob. Agents Chemother.* 56 2314–2325. 10.1128/AAC.05919-11 22314537 PMC3346669

[B24] Jakobsen T. H. Warming A. N. Vejborg R. M. Moscoso J. A. Stegger M. Lorenzen F. (2017). A broad range quorum sensing inhibitor working through sRNA inhibition. *Sci. Rep.* 7:9857. 10.1038/s41598-017-09886-8 28851971 PMC5575346

[B25] Kalia V. C. (2013). Quorum sensing inhibitors: an overview. *Biotechnol. Adv.* 31 224–245. 10.1016/j.biotechadv.2012.10.004 23142623

[B26] Kim H.-S. Lee S.-H. Byun Y. Park H.-D. (2015). 6-Gingerol reduces *Pseudomonas aeruginosa* biofilm formation and virulence via quorum sensing inhibition. *Sci. Rep.* 5:8656. 10.1038/srep08656 25728862 PMC4345325

[B27] Köhler T. Ouertatani-Sakouhi H. Cosson P. Van Delden C. (2014). QsrO a novel regulator of quorum-sensing and virulence in *Pseudomonas aeruginosa*. *PLoS One* 9:e87814. 10.1371/journal.pone.0087814 24551066 PMC3923755

[B28] Krishnan T. Yin W.-F. Chan K.-G. (2012). Inhibition of quorum sensing-controlled virulence factor production in *Pseudomonas aeruginosa* PAO1 by Ayurveda spice clove (*Syzygium aromaticum*) bud extract. *Sensors* 12 4016–4030. 10.3390/s120404016 22666015 PMC3355396

[B29] Lan L. Murray T. S. Kazmierczak B. I. He C. (2010). *Pseudomonas aeruginosa* OspR is an oxidative stress sensing regulator that affects pigment production, antibiotic resistance and dissemination during infection. *Mol. Microbiol.* 75 76–91. 10.1111/j.1365-2958.2009.06955.x 19943895 PMC2881571

[B30] LaSarre B. Federle M. J. (2013). Exploiting quorum sensing to confuse bacterial pathogens. *Microbiol. Mol. Biol. Rev.* 77 73–111. 10.1128/MMBR.00046-12 23471618 PMC3591984

[B31] Lee J. Zhang L. (2015). The hierarchy quorum sensing network in *Pseudomonas aeruginosa*. *Protein Cell* 6 26–41. 10.1007/s13238-014-0100-x 25249263 PMC4286720

[B32] Lee J.-H. Kim Y.-G. Cho H. S. Ryu S. Y. Cho M. H. Lee J. (2014). Coumarins reduce biofilm formation and the virulence of *Escherichia coli* O157: H7. *Phytomedicine* 21 1037–1042. 10.1016/j.phymed.2014.04.008 24837471

[B33] Lin Chua S. Liu Y. Li Y. Jun Ting H. Kohli G. S. Cai Z. (2017). Reduced intracellular c-di-GMP content increases expression of quorum sensing-regulated genes in *Pseudomonas aeruginosa*. *Front. Cell. Infect. Microbiol.* 7:451. 10.3389/fcimb.2017.00451 29090193 PMC5650985

[B34] Lyczak J. B. Cannon C. L. Pier G. B. (2000). Establishment of *Pseudomonas aeruginosa* infection: lessons from a versatile opportunist1. *Microbes Infect.* 2 1051–1060. 10.1016/S1286-4579(00)01259-4 10967285

[B35] McManus A. Mason A. McManus W. Pruitt B. (1985). Twenty-five year review of*Pseudomonas aeruginosa* bacteremia in a burn center. *Eur. J. Clin. Microbiol.* 4 219–223. 10.1007/BF02013601 3924612

[B36] Mulcahy L. R. Isabella V. M. Lewis K. (2014). *Pseudomonas aeruginosa* biofilms in disease. *Microb. Ecol.* 68 1–12. 10.1007/s00248-013-0297-x 24096885 PMC3977026

[B37] Nicodeme M. Grill J. P. Humbert G. Gaillard J. L. (2005). Extracellular protease activity of different *Pseudomonas* strains: dependence of proteolytic activity on culture conditions. *J. Appl. Microbiol.* 99 641–648. 10.1111/j.1365-2672.2005.02634.x 16108806

[B38] Obritsch M. D. Fish D. N. MacLaren R. Jung R. (2005). Nosocomial infections due to multidrug-resistant *Pseudomonas aeruginosa*: epidemiology and treatment options. *Pharmacotherapy* 25 1353–1364. 10.1592/phco.2005.25.10.1353 16185180

[B39] Ouyang J. Sun F. Feng W. Sun Y. Qiu X. Xiong L. (2016). Quercetin is an effective inhibitor of quorum sensing, biofilm formation and virulence factors in *Pseudomonas aeruginosa*. *J. Appl. Microbiol.* 120 966–974. 10.1111/jam.13073 26808465

[B40] Rasamiravaka T. El Jaziri M. (2016). Quorum-sensing mechanisms and bacterial response to antibiotics in *P. aeruginosa*. *Curr. Microbiol.* 73 747–753. 10.1007/s00284-016-1101-1 27449213

[B41] Rasmussen T. B. Bjarnsholt T. Skindersoe M. E. Hentzer M. Kristoffersen P. Köte M. (2005). Screening for quorum-sensing inhibitors (QSI) by use of a novel genetic system, the QSI selector. *J. Bacteriol.* 187 1799–1814. 10.1128/JB.187.5.1799-1814.2005 15716452 PMC1063990

[B42] Reen F. J. Gutiérrez-Barranquero J. A. Parages M. L. (2018). Coumarin: a novel player in microbial quorum sensing and biofilm formation inhibition. *Appl. Microbiol. Biotechnol.* 102 2063–2073. 10.1007/s00253-018-8787-x 29392389 PMC5814477

[B43] Rinaldo S. Paiardini A. Stelitano V. Brunotti P. Cervoni L. Fernicola S. (2015). Structural basis of functional diversification of the HD-GYP domain revealed by the *Pseudomonas aeruginosa* PA4781 protein, which displays an unselective bimetallic binding site. *J. Bacteriol.* 197 1525–1535. 10.1128/JB.02606-14 25691523 PMC4372746

[B44] Römling U. Galperin M. Y. Gomelsky M. (2013). Cyclic di-GMP: the first 25 years of a universal bacterial second messenger. *Microbiol. Mol. Biol. Rev.* 77 1–52. 10.1128/MMBR.00043-12 23471616 PMC3591986

[B45] Rumbaugh K. P. Griswold J. A. Iglewski B. H. Hamood A. N. (1999). Contribution of quorum sensing to the virulence of*Pseudomonas aeruginosa* in burn wound infections. *Infect. Immun.* 67 5854–5862. 10531240 10.1128/iai.67.11.5854-5862.1999 PMC96966

[B46] Ryan R. P. Lucey J. O’donovan K. McCarthy Y. Yang L. Tolker-Nielsen T. (2009). HD-GYP domain proteins regulate biofilm formation and virulence in *Pseudomonas aeruginosa*. *Environ. Microbiol* 11 1126–1136. 10.1111/j.1462-2920.2008.01842.x 19170727

[B47] Rybtke M. T. Jensen P. O. Hoiby N. Givskov M. Tolker-Nielsen T. Bjarnsholt T. (2011). The implication of *Pseudomonas aeruginosa* biofilms in infections. *Inflamm. Allergy Drug Targets* 10 141–157. 10.2174/187152811794776222 21314623

[B48] Sen C. K. Gordillo G. M. Roy S. Kirsner R. Lambert L. Hunt T. K. (2009). Human skin wounds: a major and snowballing threat to public health and the economy. *Wound Repair Regen.* 17 763–771. 10.1111/j.1524-475X.2009.00543.x 19903300 PMC2810192

[B49] Shih P.-C. Huang C.-T. (2002). Effects of quorum-sensing deficiency on *Pseudomonas aeruginosa* biofilm formation and antibiotic resistance. *J. Antimicrob. Chemother.* 49 309–314. 10.1093/jac/49.2.309 11815572

[B50] Smith R. S. Iglewski B. H. (2003). P. *aeruginosa quorum-sensing systems and virulence*. *Curr. Opin. Microbiol.* 6 56–60. 10.1016/S1369-5274(03)00008-0 12615220

[B51] Stelitano V. Giardina G. Paiardini A. Castiglione N. Cutruzzolà F. Rinaldo S. (2013). C-di-GMP hydrolysis by *Pseudomonas aeruginosa* HD-GYP phosphodiesterases: analysis of the reaction mechanism and novel roles for pGpG. *PLoS One* 8:e74920. 10.1371/journal.pone.0074920 24066157 PMC3774798

[B52] Strateva T. Mitov I. (2011). Contribution of an arsenal of virulence factors to pathogenesis of *Pseudomonas aeruginosa* infections. *Ann. Microbiol.* 61 717–732. 10.1007/s13213-011-0273-y

[B53] Ueda A. Wood T. K. (2009). Connecting quorum sensing, c-di-GMP, pel polysaccharide, and biofilm formation in *Pseudomonas aeruginosa* through tyrosine phosphatase TpbA (PA3885). *PLoS Pathog.* 5:e1000483. 10.1371/journal.ppat.1000483 19543378 PMC2691606

[B54] Ünal S. Ersoz G. Demirkan F. Arslan E. Tütüncü N. Sari A. (2005). Analysis of skin-graft loss due to infection: infection-related graft loss. *Ann. Plast. Surg.* 55 102–106. 10.1097/01.sap.0000164531.23770.60 15985801

[B55] Van Acker H. De Canck E. Van Nieuwerburgh F. Sass A. Deforce D. Nelis H. J. (2014). The BCESM genomic region contains a regulator involved in quorum sensing and persistence in Burkholderia cenocepacia J2315. *Future Microbiol.* 9 845–860. 10.2217/fmb.14.54 25156374

[B56] Van Delden C. Iglewski B. H. (1998). Cell-to-cell signaling and *Pseudomonas aeruginosa* infections. *Emerg. Infect. Dis.* 4 551. 9866731 10.3201/eid0404.980405 PMC2640238

[B57] Vandecandelaere I. Depuydt P. Nelis H. J. Coenye T. (2014). Protease production by Staphylococcus epidermidis and its effect on Staphylococcus aureus biofilms. *Pathog. Dis.* 70 321–331. 10.1111/2049-632X.12133 24436195

[B58] Wagner V. E. Bushnell D. Passador L. Brooks A. I. Iglewski B. H. (2003). Microarray analysis of *Pseudomonas aeruginosa* quorum-sensing regulons: effects of growth phase and environment. *J. Bacteriol.* 185 2080–2095. 10.1128/JB.185.7.2080-2095.2003 12644477 PMC151498

[B59] Waters C. M. Bassler B. L. (2005). Quorum sensing: cell-to-cell communication in bacteria. *Annu. Rev. Cell Dev. Biol.* 21 319–346. 10.1146/annurev.cellbio.21.012704.131001 16212498

[B60] Winsor G. L. Griffiths E. J. Lo R. Dhillon B. K. Shay J. A. Brinkman F. S. (2015). Enhanced annotations and features for comparing thousands of *Pseudomonas* genomes in the *Pseudomonas* genome database. *Nucleic Acids Res.* 44 D646–D653. 10.1093/nar/gkv1227 26578582 PMC4702867

[B61] Wu H. Song Z. Hentzer M. Andersen J. B. Molin S. Givskov M. (2004). Synthetic furanones inhibit quorum-sensing and enhance bacterial clearance in *Pseudomonas aeruginosa* lung infection in mice. *J. Antimicrob. Chemother.* 53 1054–1061. 10.1093/jac/dkh223 15117922

[B62] Zeng Z. Qian L. Cao L. Tan H. Huang Y. Xue X. (2008). Virtual screening for novel quorum sensing inhibitors to eradicate biofilm formation of *Pseudomonas aeruginosa*. *Appl. Microbiol. Biotechnol.* 79:119. 10.1007/s00253-008-1406-5 18330563

[B63] Zhang S. Liu N. Liang W. Han Q. Zhang W. Li C. (2017). Quorum sensing-disrupting coumarin suppressing virulence phenotypes in vibrio splendidus. *Appl. Microbiol. Biotechnol.* 101 3371–3378. 10.1007/s00253-016-8009-3 27942756

