# Supplementary material for: Coumarin Reduces Virulence and Biofilm Formation in Pseudomonas aeruginosa by Affecting Quorum Sensing, Type III Secretion and C-di-GMP Levels
Source: Front Microbiol. 2018 Aug 21;9:1952. doi: 10.3389/fmicb.2018.01952 (PMC6110822; doi:10.3389/fmicb.2018.01952)
Supplement: Supplementary file 1 [file Data_Sheet_1.PDF]

Figure S1. Growth curves (planktonic cultures) of the different *P. aeruginosa* strains, obtained in the absence (ctrl) or presence of coumarin (1 mM or 2 mM). Growth was measured by determining optical density at 450 nm every two hours, for up to 24 hours.

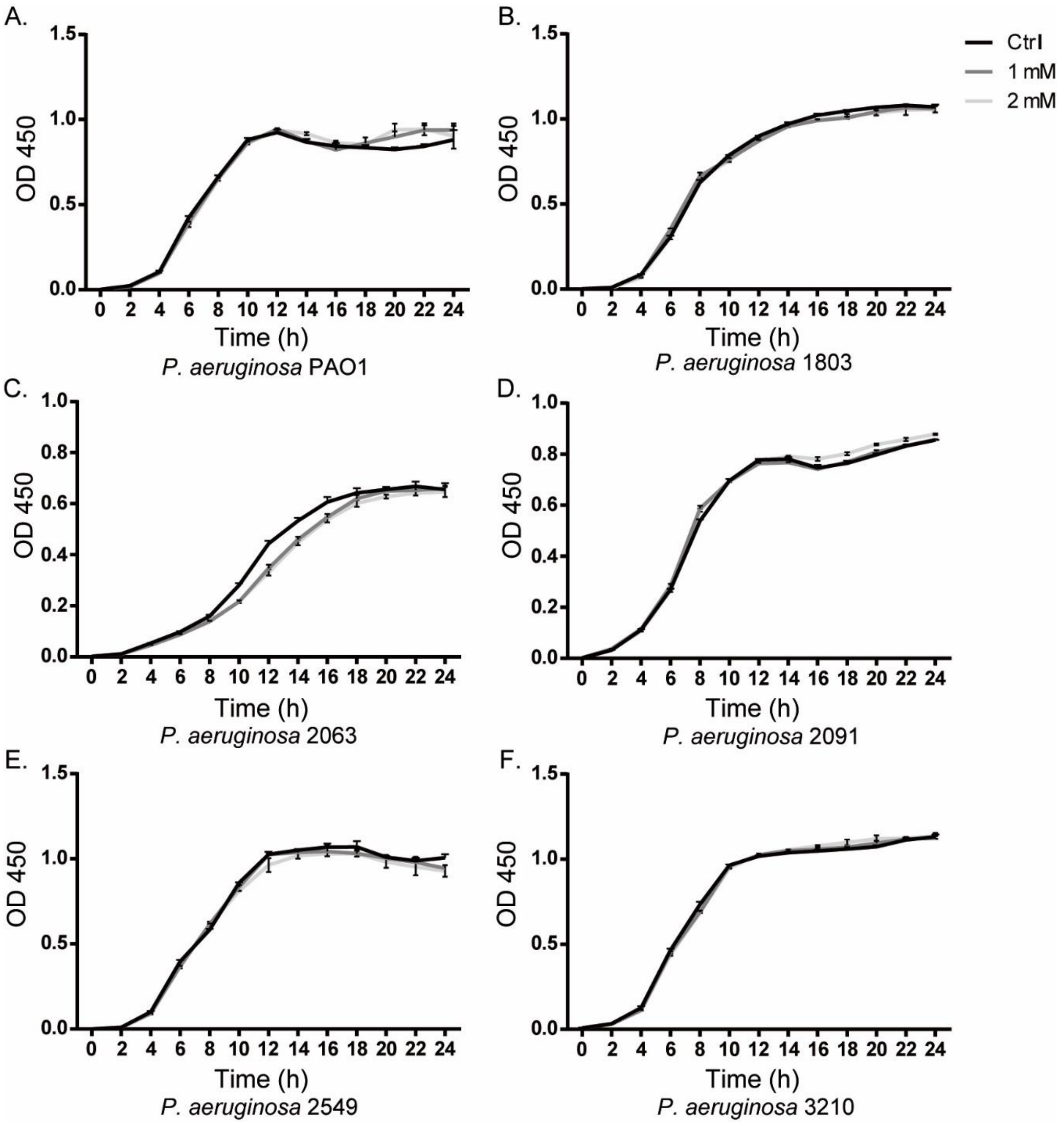

Table S1. Genes that are significantly down- or upregulated (cut-off: 1.5-fold change, FDR p-value < 0.05) in *P. aeruginosa* PAO1 planktonic cells following coumarin treatment (2 mM, 1h).

Locus tag	Gene name	Description	Fold changes
**Down-regulated genes (46)**			
Type III secretion related (21)			
PA3841	*exoS*	Exoenzyme S	-1.69
PA0044	*exoT*	Exoenzyme T	-1.71
PA1697		ATP synthase in type III secretion system	-2.27
PA1699	*pcr1*		-2.21
PA1700	*pcr2*		-2.07
PA1703	*pcrD*	Type III secretory apparatus protein	-1.72
PA1707	*pcrH*	Regulatory protein	-1.69
PA1706	*pcrV*	Type III secretion protein	-1.69
PA1708	*popB*	Translocator protein	-1.76
PA1709	*popD*	Translocator outer membrane protein	-1.82
PA1698	*popN*	Type III secretion outer membrane protein	-2.00
PA1715	*pscB*	Type III export apparatus protein	-1.64
PA1716	*pscC*	Type III secretion outer membrane protein	-1.51
PA1719	*pscF*	Type III export protein	-1.65
PA1720	*pscG*	Type III export protein	-1.72
PA1722	*pscI*	Type III export protein	-1.53
PA1723	*pscJ*	Type III export protein	-1.51
PA1724	*pscK*	Type III export protein	-1.65
PA1696	*pscO*	Translocation protein in type III secretion	-2.31
PA1695	*pscP*	Translocation protein in type III secretion	-1.93
PA1694	*pscQ*	Translocation protein in type III secretion	-2.02
Polyamine transport related (3)			
PA3608	*potB*	Polyamine transport protein	-1.94
PA3609	*potC*	Polyamine transport protein	-2.27
PA3610	*potD*	Polyamine transport protein	-2.19
Two-component regulator system (4)			
PA4774			-3.51
PA4775			-2.54
PA4776	*pmrA*		-2.51
PA4777	*pmrB*		-2.33
Others (9)			
PA1321	*cyoE*	Cytochrome o ubiquinol oxidase	-1.63
PA1838	*cysI*	Sulfite reductase	-1.70
PA1634	*kdpB*	Potassium-transporting ATPase	-1.84
PA2825	*ospR*	Oxidative stress sensing regulator	-1.91
PA2826		Glutathione peroxidase	-2.06
PA3677	*MexJ*	Efflux pump	-1.69

PA5365	*phoU*	Phosphate uptake regulatory protein	-1.50
PA1905	*phzG2*	Probable pyridoxamine 5'-phosphate oxidase	-50.75
PA4781		Cyclic di-GMP phosphodiesterase	-2.01
Unknown function (9)			
PA1228			-2.51
PA1402			-1.83
PA2283			-1.94
PA2284			-2.13
PA2285			-2.31
PA3445			-1.77
PA4359			-2.13
PA4773			-3.15
PA4782			-2.30
**Up-regulated genes (21)**			
Multi-drug efflux related (12)			
PA3719	*armR*	Antirepressor for mexr	8.35
PA0425	*mexA*	Multidrug efflux membrane fusion protein	2.39
PA0426	*mexB*	Multidrug efflux transporter	2.06
PA4599	*mexC*	Multidrug efflux membrane fusion protein	3.04
PA4598	*mexD*	Multidrug efflux transporter	3.05
PA0424	*mexR*	Multidrug resistance operon repressor	2.03
PA3721	*nalC*	A probable repressor of the TetR/AcrRfamily	1.70
PA4597	*oprJ*	Multidrug efflux outer membrane protein	3.48
PA0427	*oprM*	Multidrug efflux outer membrane protein	1.78
PA3718		Probable major facilitator superfamily transporter	3.06
PA3720		Hypothetical protein	7.41
PA4596	*esrC*	An envelope stress-regulated repressor	3.12
Other genes and genes with unknown functions (9)			
PA3126	*ibpA*	Heat-shock protein	1.52
PA4047	*ribA*	GTP cyclohydrolase II	1.74
PA1922		Probable TonB-dependent receptor	2.11
PA2930		Probable transcriptional regulator	1.64
PA4045			1.54
PA4046			1.66
PA4288		Probable transcriptional regulator	1.80
PA4837		Probable outer membrane protein precursor	1.96
PA4838			2.06

Table S2. Genes that are significantly down-regulated (cut-off: 1.5-fold change, FDR p-value < 0.05) in *P. aeruginosa* PAO1 24h-biofilm cells treated with coumarin (2 mM).

**Locus tag**	**Gene name**	**Description**	**Fold changes**
QS network (11)			
PA1432	*lasI*	AHL synthesis protein LasI	-1.76
PA3476	*rhlI*	AHL synthesis protein RhlI	-1.62
PA3477	*rhlR*	Transcriptional regulator RhlR	-1.85
PA0997	*pqsB*	PQS synthesis	-1.62
PA0998	*pqsC*		-1.62
PA2587	*pqsH*		-1.96
PA4190	*pqsL*		-1.91
PA2305	*ambB*	IQS synthesis	-2.22
PA2304	*ambC*		-2.27
PA2303	*ambD*		-2.30
PA2302	*ambE*		-1.84
major QS-regulated virulence factors (43)			
PA1249	*aprA*	Synthesis and secretion of alkaline protease	-5.06
PA1246	*aprD*		-1.93
PA1247	*aprE*		-2.34
PA1248	*aprF*		-1.83
PA1250	*aprI*		-2.48
PA2193	*hcnA*	Hydrogen cyanide synthesis	-2.75
PA2194	*hcnB*		-1.94
PA2195	*hcnC*		-1.75
PA1871	*lasA*	LasA protease	-4.93
PA3724	*lasB*	LasB elastase	-3.06
PA2570	*lecA*	Lectin	-3.21
PA4231	*pchA*	Pyochelin biosynthesis	-1.80
PA4230	*pchB*		-2.22
PA4228	*pchD*		-1.98
PA4226	*pchE*		-1.78
PA4225	*pchF*		-1.66
PA4224	*pchG*		-1.84
PA4211	*phzB1*	Phenazine biosynthesis	-2.24
PA1900	*phzB2*		-3.19
PA1901	*phzC2*		-1.93
PA4213	*phzD1*		-1.81
PA1902	*phzD2*		-1.92
PA4214	*phzE1*		-1.98
PA1903	*phzE2*		-1.97
PA4215	*phzF1*		-2.17
PA1904	*phzF2*		-2.18

PA1905	*phzG2*		-3.36
PA4217	*phzS*		-1.80
PA2386	*pvdA*	Pyoverdine biosynthesis and transport	-1.99
PA2396	*pvdF*		-1.71
PA2425	*pvdG*		-1.65
PA2413	*pvdH*		-2.12
PA2394	*pvdN*		-1.87
PA2395	*pvdO*		-1.85
PA2392	*pvdP*		-1.93
PA2426	*pvdS*		-1.72
PA2390	*pvdT*		-1.62
PA2391	*opmQ*		-1.69
PA3479	*rhlA*	Rhamnolipid production	-2.57
PA3478	*rhlB*		-2.09
PA2300	*chiC*	Chitinase	-6.93
PA4236	*katA*	Catalase	-1.56
PA4468	*sodM*	Superoxide dismutase	-2.38
Type III secretion related (18)			
PA3841	*exoS*	Exoenzyme S	-1.70
PA0044	*exoT*	Exoenzyme T	-1.57
PA1697		ATP synthase in type III secretion system	-2.18
PA1699	*pcr1*		-1.68
PA1710	*exsC*	Exoenzyme S synthesis protein C precursor	-1.99
PA1714	*exsD*	Type III secretion regulator	-1.77
PA1705	*pcrG*	Type III secretion regulator	-2.22
PA1707	*pcrH*	Regulatory protein	-2.66
PA1706	*pcrV*	Type III secretion protein	-2.06
PA1708	*popB*	Translocator protein	-2.01
PA1709	*popD*	Translocator outer membrane protein	-2.31
PA1698	*popN*	Type III secretion outer membrane protein	-2.80
PA1715	*pscB*	Type III export apparatus protein	-1.68
PA1719	*pscF*	Type III export protein	-1.82
PA1695	*pscP*	Translocation protein in type III secretion	-3.23
PA1694	*pscQ*	Translocation protein in type III secretion	-1.93
PA2808	*ptrA*	*Pseudomonas* type III repressor A	-2.00
PA1711	*exsE*	A regulator of ExsC	-1.79
C-di-GMP metabolism (1)			
PA4781		Cyclic di-GMP phosphodiesterase	-2.00
Others (121)			
PA5427	*adhA*	Alcohol dehydrogenase	-1.56
PA1337	*ansB*	Glutaminase-asparaginase	-1.68
PA5171	*arcA*	Arginine deiminase	-1.67
PA5172	*arcB*	Ornithine carbamoyltransferase	-1.66
PA5173	*arcC*	Carbamate kinase	-1.69

PA2886	*atuA*	Citronellol catabolism	-1.67
PA2887	*atuB*		-1.85
PA2888	*atuC*		-1.71
PA2889	*atuD*		-1.66
PA2890	*atuF*		-1.73
PA2891	*atuG*		-1.50
PA2003	*bdhA*	3-hydroxybutyrate dehydrogenase	-2.35
PA2052	*cynS*	Cyanate lyase	-1.73
PA2000	*dhcB*	Dehydrocarnitine CoA transferase	-1.52
PA2008	*fahA*	Fumarylacetoacetase	-1.58
PA4470	*fumC1*	Fumarate hydratase	-2.83
PA0854	*fumC2*	Fumarate hydratase	-1.56
PA1421	*gbuA*	Guanidinobutyrase	-1.58
PA2446	*gcvH2*	Glycine cleavage system protein	-1.56
PA2445	*gcvP2*		-1.64
PA2442	*gcvT2*		-1.59
PA2153	*glgB*	1,4-alpha-glucan branching enzyme	-1.77
PA2144	*glgP*	Glycogen phosphorylase	-1.60
PA2444	*glyA2*	Serine hydroxymethyltransferase	-1.63
PA5091	*hutG*	Histidine utilization	-1.63
PA5098	*hutH*		-1.62
PA5092	*hutI*		-1.69
PA5100	*hutU*		-2.03
PA4694	*ilvC*	Ketol acid reductoisomerase	-2.10
PA4695	*ilvH*	Small acetolactate synthase subunit	-1.91
PA4696	*ilvI*	Acetolactate synthase enzyme	-1.67
PA3792	*leuA*	Leucine synthesis	-2.93
PA3118	*leuB*		-2.44
PA3121	*leuC*		-4.10
PA3120	*leuD*		-3.27
PA4370	*icmP*	Insulin-cleaving metalloproteinase outer membrane protein	-1.67
PA2863	*lipH*	Lipase modulator protein	-1.53
PA4770	*lldP*	L-lactate permease	-1.82
PA2007	*maiA*	Maleylacetoacetate isomerase	-1.51
PA1927	*metE*	Methionine synthesis	-1.51
PA0546	*metK*		-1.74
PA5025	*metY*		-1.62
PA0132		Beta-alanine:pyruvate transaminase	-1.92
PA0399		Cystathionine beta-synthase	-1.81
PA1041	*wapB*	1,2-glucosyltransferase	-2.09
PA1256	*lhpO*	ABC transporter ATP-binding protein	-2.06
PA1641	*gpsA*	Glycerol-3-phosphate dehydrogenase	-1.57
PA2321		Gluconokinase	-1.57
PA2414		L-sorbosone dehydrogenase	-1.86

PA4022	*hdhA*	Hydrazone dehydrogenase	-1.76
PA4661	*pagL*	Lipid A 3-O-deacylase	-1.71
PA5058	*phaC2*	Poly(3-hydroxyalkanoic acid) synthase 2	-1.84
PA5161	*rmlB*	dTDP-D-glucose 4,6-dehydratase	-1.51
PA0849	*trxB2*	Thioredoxin reductase 2	-2.40
PA5419	*soxG*	Sarcosine oxidase gamma subunit	-1.83
PA2279	*arsC*	Arsenate reductase	-1.51
PA1863	*modA*	Molybdate-binding periplasmic protein precursor	-1.50
PA0513	*nirG*	Nitrite reductase	-1.50
PA1177	*napE*	Periplasmic nitrate reductase protein	-1.84
PA0523	*norC*	Nitric-oxide reductase subunit C	-2.39
PA0023	*qor*	Quinone oxidoreductase	-1.64
PA3531	*bfrB*	Bacterioferritin	-1.72
PA3407	*hasAp*	Heme acquisition protein	-8.13
PA3408	*hasR*	Heme uptake outer membrane receptor HasR precursor	-1.88
PA3530	*bfd*	Bacterioferritin-associated ferredoxin	-1.62
PA3676	*mexK*	Efflux pump	-1.58
PA3677	*mexJ*	Efflux pump	-1.73
PA4205	*mexG*	Membrane protein required for MexGHI-OpmD efflux	-1.71
PA4587	*ccpR*	Cytochrome c551 peroxidase precursor	-1.97
PA1318	*cyoB*	Cytochrome o ubiquinol oxidase subunit	-1.83
PA1319	*cyoC*		-2.61
PA1320	*cyoD*		-2.09
PA1321	*cyoE*	Cytochrome o ubiquinol oxidase protein	-2.04
PA4133		Cytochrome c oxidase subunit	-1.52
PA3692	*lptF*	Lipotoxon	-1.99
PA0122	*rahU*		-2.06
PA5285	*SutA*	A bacterial transcription factor	-1.85
PA0843	*plcR*	Phospholipase accessory protein	-1.63
PA4776	*pmrA*	Two-component regulator system	-1.68
PA4777	*pmrB*		-1.85
PA3790	*oprC*	Outer membrane porin precursor	-2.87
PA1777	*oprF*	Outer membrane porin precursor	-1.54
PA4067	*oprG*	Outer membrane protein precursor	-1.64
PA1178	*oprH*	Outer membrane protein H1	-1.69
PA2853	*oprI*	Outer membrane lipoprotein	-2.08
PA4761	*dnaK*	DnaK protein	-1.56
PA4385	*groEL*	GroEL protein	-2.06
PA4386	*groES*	GroES protein	-1.78
PA4762	*grpE*	Heat shock protein	-1.57
PA5053	*hslV*	Heat shock protein	-1.57
PA1596	*htpG*	Heat shock protein	-1.93
PA0852	*cbpD*	Chitin-binding protein	-4.54
PA0139	*ahpC*	Alkyl hydroperoxide reductase subunit C	-1.67

PA3550	*algF*	Alginate o-acetyltransferase	-1.66
PA0723	*coaB*	Coat protein B of bacteriophage Pf1	-1.91
PA2717	*cpo*	Chloroperoxidase precursor	-1.63
PA0694	*exbD2*	Transport protein	-2.28
PA4306	*flp*	Type IVb pilin, Flp	-2.03
PA0867	*mliC*	Membrane-bound lysozyme inhibitor of c-type lysozyme	-1.69
PA4614	*mscL*	Conductance mechanosensitive channel	-1.87
PA0766	*mucD*	Serine protease mucd precursor	-1.52
PA0059	*osmC*	Osmotically inducible protein	-2.19
PA4876	*osmE*	Osmotically inducible lipoprotein	-2.50
PA0678	*HxcU*	Alkaline phosphatase secretion related	-2.15
PA0683	*HxcY*		-2.89
PA0779	*asrA*	ATP-dependent protease	-1.52
PA1245	*AprX*		-3.34
PA1259	*LhpH*		-1.51
PA1657	*HsiB2*		-2.04
PA1658	*hsiC2*		-1.67
PA1665	*Fha2*		-1.70
PA1668	*DotU2*		-1.58
PA1830		Lon protease	-1.58
PA3891	*opuC*	ABC transporter	-1.75
PA4916	*nrtR*	Nudix-related transcriptional regulator	-1.51
PA0355	*pfpI*	Protease PfpI	-1.97
PA4590	*pra*	Protein activator	-2.02
PA4305	*rcpC*		-1.69
PA3049	*rmf*	Ribosome modulation factor	-1.55
PA4865	*ureA*	Urease gamma subunit	-2.30
PA4868	*ureC*	Urease alpha subunit	-1.70
Genes with general predicted or unknown functions (205)			
PA3672		Probable ATP-binding component of ABC transporte	-1.55
PA4223		Probable ATP-binding component of ABC transporter	-1.75
PA0400		Probable cystathionine gamma-lyase	-1.75
PA0656		Probable HIT family protein	-1.58
PA4131		Probable iron-sulfur protein	-2.30
PA1131		Probable major facilitator superfamily (MFS) transporter	-1.68
PA4144		Probable outer membrane protein precursor	-2.79
PA4171		Probable protease	-2.32
PA4143		Probable toxin transporter	-3.21
PA0236		Probable transcriptional regulator	-1.96
PA1403		Probable transcriptional regulator	-1.51
PA2588		Probable transcriptional regulator	-1.89
PA4341		Probable transcriptional regulator	-1.86
PA3963a		Probable transporter	-2.43
PA1737		Probable 3-hydroxyacyl-CoA dehydrogenase	-1.56

PA1869	Probable acyl carrier protein	-2.71
PA2815	Probable acyl-CoA dehydrogenase	-1.63
PA2158	Probable alcohol dehydrogenase (Zn-dependent)	-1.52
PA0366	Probable aldehyde dehydrogenase	-1.51
PA5097	Probable amino acid permease	-1.62
PA1617	Probable AMP-binding enzyme	-1.69
PA5094	Probable ATP-binding component of ABC transporter	-1.51
PA5096	Probable binding protein component of ABC transporter	-2.06
PA2069	Probable carbamoyl transferase	-3.40
PA1251	Probable chemotaxis transducer	-1.96
PA0459	Probable ClpA/B protease ATP binding subunit	-1.59
PA0223	Probable dihydrodipicolinate synthetase	-2.67
PA3940	Probable DNA binding protein	-1.53
PA2086	Probable epoxide hydrolase	-1.85
PA2165	Probable glycogen synthase	-1.87
PA2160	Probable glycosyl hydrolase	-2.14
PA2162	Probable glycosyl hydrolase	-1.83
PA2164	Probable glycosyl hydrolase	-2.27
PA5093	Probable histidine/phenylalanine ammonia-lyase	-1.54
PA1202	Probable hydrolase	-1.71
PA2067	Probable hydrolase	-2.38
PA2698	Probable hydrolase	-1.65
PA2068	Probable major facilitator superfamily (MFS) transporter	-2.30
PA3441	Probable molybdopterin-binding protein	-1.72
PA4078	Probable nonribosomal peptide synthetase	-1.79
PA4172	Probable nuclease	-1.68
PA1875	Probable outer membrane protein precursor	-1.71
PA0147	Probable oxidoreductase	-1.52
PA1127	Probable oxidoreductase	-1.51
PA2592	Probable periplasmic spermidine/putrescine-binding protein	-1.80
PA3315	Probable permease of ABC transporter	-1.55
PA5095	Probable permease of ABC transporter	-1.59
PA3913	Probable protease	-1.88
PA4142	Probable secretion protein	-4.35
PA1344	Probable short-chain dehydrogenase	-1.78
PA2142	Probable short-chain dehydrogenase	-1.64
PA4098	Probable short-chain dehydrogenase	-1.84
PA2411	Probable thioesterase	-1.58
PA1285	Probable transcriptional regulator	-1.61
PA2096	Probable transcriptional regulator	-1.59
PA2312a	Probable transcriptional regulator	-1.54
PA3965	Probable transcriptional regulator	-1.72
PA4023	Probable transport protein	-1.57

PA2135	Probable transporter	-1.55
PA2393	Putative dipeptidase	-1.94
PA0039	Hypothetical protein	-1.62
PA0050	Hypothetical protein	-1.61
PA0060	Hypothetical protein	-1.53
PA0062	Hypothetical protein	-1.62
PA0116	Hypothetical protein	-1.55
PA0187	Hypothetical protein	-1.79
PA0188	Hypothetical protein	-1.83
PA0250	Hypothetical protein	-1.54
PA0269	Hypothetical protein	-2.26
PA0270	Hypothetical protein	-1.99
PA0271	Hypothetical protein	-1.71
PA0307	Hypothetical protein	-1.51
PA0526	Hypothetical protein	-1.72
PA0529	Hypothetical protein	-1.65
PA0572	Hypothetical protein	-2.47
PA0741	Hypothetical protein	-1.97
PA0851	Hypothetical protein	-1.60
PA1123	Hypothetical protein	-2.18
PA1135	Hypothetical protein	-1.53
PA1198	Hypothetical protein	-1.51
PA1244	Hypothetical protein	-1.71
PA1323	Hypothetical protein	-2.22
PA1324	Hypothetical protein	-2.30
PA1353	Hypothetical protein	-1.60
PA1404	Hypothetical protein	-2.43
PA1414	Hypothetical protein	-1.50
PA1478	Hypothetical protein	-1.57
PA1592	Hypothetical protein	-1.53
PA1597	Hypothetical protein	-1.59
PA1784	Hypothetical protein	-1.54
PA1852	Hypothetical protein	-1.72
PA1870	Hypothetical protein	-1.81
PA1906	Hypothetical protein	-1.62
PA1913	Hypothetical protein	-1.67
PA2004	Hypothetical protein	-2.25
PA2026	Hypothetical protein	-2.52
PA2027	Hypothetical protein	-4.67
PA2030	Hypothetical protein	-1.94
PA2031	Hypothetical protein	-2.02
PA2033	Hypothetical protein	-2.10
PA2066	Hypothetical protein	-1.88
PA2134	Hypothetical protein	-2.79

PA2141	Hypothetical protein	-2.18
PA2142a	Hypothetical protein	-1.58
PA2143	Hypothetical protein	-1.98
PA2381	Hypothetical protein	-2.83
PA2146		-2.97
PA2148		-1.75
PA2149		-2.16
PA2154		-1.51
PA2159		-1.99
PA2161		-2.44
PA2163		-2.10
PA2166		-2.49
PA2169		-2.20
PA2171		-2.03
PA2172		-1.58
PA2173		-1.55
PA2176		-1.54
PA2178		-1.50
PA2180		-1.72
PA2184		-2.05
PA2190		-2.71
PA2197		-1.80
PA2274		-2.33
PA2383		-1.66
PA2384		-2.75
PA2412		-1.95
PA2415		-1.55
PA2422		-1.56
PA2427		-1.69
PA2433		-2.23
PA2448		-1.97
PA2453		-1.78
PA2566		-1.60
PA2747		-1.77
PA2785		-1.80
PA2860		-1.72
PA2927		-1.51
PA3041		-1.73
PA3042		-1.58
PA3051		-1.57
PA3119		-4.16
PA3123		-1.51
PA3130		-1.55
PA3273		-1.78

PA3274	-1.59
PA3275	-1.72
PA3313	-1.82
PA3314	-1.64
PA3370	-1.62
PA3371	-1.60
PA3412	-2.18
PA3520	-2.86
PA3532	-1.84
PA3691	-1.91
PA3734	-1.72
PA3784	-1.77
PA3785	-2.16
PA3786	-1.52
PA3791	-1.93
PA3819	-1.65
PA3904	-1.58
PA3906	-1.82
PA3907	-1.51
PA3908	-1.91
PA4129	-1.60
PA4132	-1.70
PA4139	-3.49
PA4141	-7.57
PA4311	-1.51
PA4313a	-1.65
PA4346	-1.51
PA4352	-1.55
PA4384	-1.84
PA4467	-1.94
PA4469	-2.86
PA4471	-1.62
PA4570	-2.29
PA4573	-1.52
PA4578	-1.58
PA4607	-1.95
PA4702	-1.53
PA4738	-1.87
PA4739	-1.90
PA4773	-2.32
PA4774	-3.28
PA4775	-2.18
PA4782	-3.66
PA4866	-1.72

PA4874	-1.66
PA4877	-1.73
PA4925	-1.51
PA5061	-1.70
PA5101	-1.52
PA5178	-1.61
PA5212	-1.55
PA5219	-1.58
PA5220	-2.28
PA5286	-1.51
PA5424	-1.65
PA5460	-4.02
PA5461	-1.59
PA5481	-1.86
PA5482	-1.89

Table S3. Genes that are significantly up-regulated (cut-off: 1.5-fold change, FDR p-value < 0.05) in *P. aeruginosa* PAO1 24h-biofilm cells treated with coumarin (2 mM).

Locus tag	Gene name	Description	Fold changes
QS regulator (1)			
PA2226	*qsrO*	QS negative regulator	3.70
C-di-GMP metabolism (1)			
PA3885	*tpbA*	Protein tyrosine phosphatase TpbA	3.95
Others (85)			
PA2513	*antB*	Anthranilate dioxygenase small subunit	2.03
PA2514	*antC*	Anthranilate dioxygenase reductase	1.85
PA0866	*aroP2*	Aromatic amino acid transport protein	1.98
PA2507	*catA*	Catechol 1,2-dioxygenase	1.81
PA2508	*catB*	Muconate cycloisomerase I	1.72
PA2509	*catC*	Muconolactone delta-isomerase	1.62
PA0286	*desA*	Delta-9 fatty acid desaturase, DesA	1.81
PA4888	*desB*	Acyl-CoA delta-9-desaturase, DesB	1.56
PA3603	*dgkA*	Diacylglycerol kinase	1.91
PA4728	*folK*	2-amino-4-hydroxy-6-hydroxymethyldihydropteridine pyrophosphokinase	1.53
PA1384	*galE*	UDP-glucose 4-epimerase	1.86
PA3152	*hisH2*	Glutamine amidotransferase	1.68
PA4406	*lpxC*	UDP-3-O-acyl-N-acetylglucosamine deacetylase	1.64
PA0208	*mdcA*	Malonate decarboxylase	2.21
PA0210	*mdcC*		2.89
PA0211	*mdcD*		2.97
PA0212	*mdcE*		3.28
PA4898	*opdK*	Histidine porin OpdK	1.96
PA0216	*madM*	Malonate transporter	1.77
PA0603	*agtA*	4-aminobutyrate and 5-aminovalerate uptake	2.70
PA0604	*agtB*		1.97
PA0605	*agtC*		2.08
PA0606	*agtD*		1.70
PA0654	*speD*	S-adenosylmethionine decarboxylase proenzyme	2.34
PA0298	*spuB*	Glutamylpolyamine synthetase	1.81
PA5118	*thiI*	Thiazole biosynthesis protein	1.67
PA2515	*xylL*	Cis-1,2-dihydroxycyclohexa-3,4-diene carboxylate dehydrogenase	2.55
PA2518	*xylX*	Toluate 1,2-dioxygenase alpha subunit	1.52
PA2516	*xylZ*	Toluate 1,2-dioxygenase electron transfer component	1.78
PA2082	*kynR*	Lrp/AsnC-type transcriptional regulator	1.92
PA1175	*napD*	Protein of periplasmic nitrate reductase	1.52
PA3879	*narL*	Two-component response regulator NarL	1.64

PA4745	*nusA*	N utilization substance protein A	1.69
PA1779		Assimilatory nitrate reductase	1.66
PA5291	*betT2*	Glycine betaine-specific importer	1.77
PA1260	*lhpP*	ABC transporter periplasmic-binding protein,	1.97
PA0455	*dbpA*	RNA helicase DbpA	1.54
PA5239	*rho*	Transcription termination factor Rho	1.85
PA4242	*rpmJ*	50S ribosomal protein L36	1.59
PA4264	*rpsJ*	30S ribosomal protein S10	1.74
PA4563	*rpsT*	30S ribosomal protein S20	2.11
PA0579	*rpsU*	30S ribosomal protein S21	1.98
PA4727	*pcnB*	Poly(A) polymerase	1.59
PA4281	*sbcD*	Exonuclease	1.77
PA2619	*infA*	Initiation factor	1.86
PA4743	*rbfA*	Ribosome-binding factor A	1.68
PA4159	*fepB*	Ferrienterobactin-binding periplasmic protein precursor	1.66
PA0472	*fiuI*		1.64
PA3410	*hasI*		1.64
PA3899	*fecI*		1.52
PA4156	*fvbA*		3.53
PA5531	*tonB1*	(Ferri)pyoverdine signal transduction	1.68
PA1099	*fleR*	Two-component response regulator	1.63
PA1085	*flgJ*	Flagellar protein	1.70
PA1104	*fliI*	Flagellum-specific ATP synthase	1.67
PA1105	*fliJ*	Flagellar protein flij	1.57
PA4649	*cupE2*	Pilin subunit	1.74
PA4650	*cupE3*	Pilin subunit	2.06
PA0408	*pilG*	Twitching motility protein	2.37
PA4554	*pilY1*	Type 4 fimbrial biogenesis protein PilY1	1.67
PA3719	*armR*	Antirepressor for MexR	3.60
PA3721	*nalC*		1.74
PA4596	*esrC*	Repressor of the mexCD-oprJ multidrug efflux	2.05
PA5512	*mifS*	NtrC-like transcriptional regulators	1.59
PA0087	*tssE1*		1.69
PA0172	*siaA*		1.51
PA0845	*cerN*		1.54
PA1509	*tplEi*	Immunity protein	1.54
PA1510	*tplE*	Type 6 PGAP1-like effector,	1.83
PA4033	*mucE*		2.10
PA4674	*higA*	Antitoxin	1.79
PA4844	*ctpL*		1.58
PA5325	*sphA*		1.59
PA2258	*ptxR*	Transcriptional regulator	1.73
PA4034	*aqpZ*	Aquaporin Z	2.34
PA3266	*capB*	Cold acclimation protein B	2.02

PA0993	*cupC2*	Chaperone	2.92
PA0199	*exbD1*	Transport protein ExbD	1.88
PA5267	*hcpB*	Secreted protein Hcp	1.58
PA3007	*lexA*	Repressor protein	1.61
PA0320	*carO*	Calcium-regulated OB-fold protein	2.22
PA0327	*carP*	Calcium-regulated beta-propeller protein	1.56
PA0930		Two-component sensor	1.61
PA4276	*secE*	Secretion protein	1.69
PA3153	*wzx*	O-antigen translocase	1.96
Genes with general predicted or unknown functions (147)			
PA0214		Probable acyl transferase	2.22
PA4979		Probable acyl-CoA dehydrogenase	1.59
PA1601		Probable aldehyde dehydrogenase	1.6
PA3865a		Probable amino acid binding protein	1.79
PA5503		Probable ATP-binding component of ABC transporter	1.59
PA2840		Probable ATP-dependent RNA helicase	2.25
PA1541		Probable drug efflux transporter	2.49
PA4980		Probable enoyl-coa hydratase/isomerase	2.25
PA1385		Probable glycosyl transferase	2.53
PA3573		Probable major facilitator superfamily (MFS) transporter	1.7
PA1602		Probable oxidoreductase	1.79
PA1739		Probable oxidoreductase	1.6
PA4889		Probable oxidoreductase Add	1.51
PA0295		Probable periplasmic polyamine binding protein	1.62
PA3189		Probable permease of ABC sugar transporter	1.67
PA0733		Probable pseudouridylate synthase	1.74
PA4896		Probable sigma-70 factor, ECF subfamily	1.7
PA0942		Probable transcriptional regulator	1.93
PA1283		Probable transcriptional regulator	1.89
PA2100		Probable transcriptional regulator	1.5
PA2220		Probable transcriptional regulator	1.52
PA2221		Probable transcriptional regulator	2.38
PA2276		Probable transcriptional regulator	1.61
PA2577		Probable transcriptional regulator	1.61
PA2957		Probable transcriptional regulator	1.59
PA3067		Probable transcriptional regulator	1.69
PA3341		Probable transcriptional regulator	1.64
PA5437		Probable transcriptional regulator	2.45
PA0730		Probable transferase	2.66
PA2480		Probable two-component sensor	1.56
PA3206		Probable two-component sensor	1.58
PA2666		Probable 6-pyruvoyl tetrahydrobiopterin synthase	2.08
PA0224		Probable aldolase	1.6
PA1964		Probable ATP-binding component of ABC transporter	1.66

PA2104	Probable cysteine synthase	1.95
PA1391	Probable glycosyl transferase	1.55
PA1569	Probable major facilitator superfamily (MFS) transporter	2
PA5370	Probable major facilitator superfamily (MFS) transporter	1.59
PA1225	Probable NAD(P)H dehydrogenase	2.05
PA4514	Probable outer membrane receptor for iron transport	1.71
PA4167	Probable oxidoreductase	4.29
PA2711	Probable periplasmic spermidine/putrescine-binding protein	1.78
PA3188	Probable permease of ABC sugar transporter	1.68
PA0975	Probable radical activating enzyme	1.64
PA0491	Probable transcriptional regulator	2.19
PA0876	Probable transcriptional regulator	1.78
PA1836	Probable transcriptional regulator	1.52
PA2047	Probable transcriptional regulator	1.66
PA2497	Probable transcriptional regulator	1.58
PA3458	Probable transcriptional regulator	2.23
PA4288	Probable transcriptional regulator	2.53
PA2479	Probable two-component response regulator	1.65
PA0006		1.61
PA0013		1.54
PA0040		1.57
PA0046		1.61
PA0069		1.69
PA0128		1.54
PA0142		1.87
PA0201		2.25
PA0209		3.27
PA0234		1.59
PA0239		1.63
PA0388		1.78
PA0457		1.52
PA0466		1.88
PA0539		1.57
PA0560		1.58
PA0561		1.7
PA0589		1.53
PA0596		1.63
PA0734		1.57
PA0758		1.71
PA0805		1.86
PA0874		2.32
PA0924		2.41
PA0976		1.75

PA0986	3.6
PA1030	1.77
PA1090	1.62
PA1116	1.64
PA1170	1.69
PA1392	1.56
PA1415	1.64
PA1428	1.73
PA1539	1.7
PA1542	2.58
PA1639	1.52
PA1741	1.61
PA1743	2.08
PA1744	2.08
PA1768	1.59
PA1769	1.61
PA1865	2.08
PA2048	1.86
PA2049	1.68
PA2101	1.6
PA2225	2.02
PA2228	1.57
PA2282	1.65
PA2288	1.72
PA2418	1.79
PA2439	1.7
PA2440	2.19
PA2457	1.67
PA2481	1.56
PA2501	1.53
PA2763a	2
PA2767	2.06
PA2910	2.11
PA2929	1.98
PA2941	1.8
PA3056	1.52
PA3057	1.67
PA3237	1.78
PA3292	1.6
PA3390	2.25
PA3413	2.86
PA3414	2.45
PA3424	1.57
PA3489	1.78

PA3720	2.21
PA3979	1.83
PA4087	2.12
PA4278	1.83
PA4338	2.32
PA4353	1.71
PA4517	4.05
PA4518	1.56
PA4523	1.66
PA4574	1.68
PA4582	3.06
PA4583	3.37
PA4584	2.1
PA4630	1.64
PA4658	1.67
PA4746	1.98
PA4817	1.66
PA4849	1.5
PA4881	3.21
PA5087	1.66
PA5209	1.65
PA5248	1.62
PA5284	2.09
PA5404	2.02
PA5406	1.79
PA5492	1.73
